# Green qualities in the neighbourhood and mental health – results from a longitudinal cohort study in Southern Sweden

**DOI:** 10.1186/1471-2458-12-337

**Published:** 2012-05-08

**Authors:** Matilda Annerstedt, Per-Olof Östergren, Jonas Björk, Patrik Grahn, Erik Skärbäck, Peter Währborg

**Affiliations:** 1 Faculty of Landscape Planning, Horticulture, and Agricultural Sciences, Department of Work Science, Business Economics and Environmental Psychology, Swedish University of Agricultural Sciences, Alnarp, Sweden; 2 Division of Social Medicine and Global Health, Department of Clinical Sciences Malmö, Lund University, Lund, Sweden; 3 Competence Centre for Clinical Research, Lund University Hospital, Lund, Sweden

**Keywords:** Environment, Population health, Stress, Salutogenic, GIS, Landscape assessment, Synergistic effect, Physical activity, GHQ12

## Abstract

**Background:**

Poor mental health is a major issue worldwide and causality is complex. For diseases with multifactorial background synergistic effects of person- and place- factors can potentially be preventive. Nature is suggested as one such positive place-factor. In this cohort study we tested the effect of defined green qualities (*Serene, Space, Wild, Culture, Lush*) in the environment at baseline on mental health at follow-up. We also studied interaction effects on mental health of those place factors and varied person factors (financial stress, living conditions, and physical activity).

**Methods:**

Data on person factors were extracted from a longitudinal (years 1999/2000 and 2005) population health survey (n = 24945). The participants were geocoded and linked to data on green qualities from landscape assessments, and stored in the Geographical Information System (GIS). Crude odds ratios (OR) and 95% confidence intervals (CI) were calculated, and multivariate logistic analyses were performed.

**Results:**

Mental health was not affected by access to the chosen green qualities, neither in terms of amount nor in terms of any specific quality. However, we found a reduced risk for poor mental health at follow-up among women, through a significant interaction effect between physical activity and access to the qualities Serene or Space. For men the tendencies were similar, though not significant. Regarding the other three green qualities, as well as amount of qualities, no statistically certain synergistic effects were found. Likewise, no significant synergies were detected between green qualities and the other person-factors. Only advanced exercise significantly reduced the risk for poor mental health among women, but not for men, compared to physical inactivity.

**Conclusions:**

The results do not directly support the hypothesis of a preventive mental health effect by access to the green qualities. However, the additive effect of serene nature to physical activity contributed to better mental health at follow-up. This tendency was equal for both sexes, but statistically significant only for women.

Objective landscape assessments may be important in detangling geographic determinants of health. This study stresses the importance of considering interaction effects when dealing with disorders of multifactorial background.

## Background

In spite of general health improvements and an increasing average lifespan in most European countries, the prevalence of mental disorders is rising cross-nationally [1]. Mental and behavioural disorders are estimated to account for 12% of the global burden of disease [2]. Gender differences reported throughout the world remain partly etiologically unclear, but the 2:1 ratio (women:men) for major depression seems to be rather consistent cross-culturally [3].

According to a survey performed in Southern Sweden in 2005 the prevalence of mental ill health, based on self-assessment methods, was 15% among men, and 21% among women (2004) [4]. This is mirrored by a growing number of people who are on sick leave due to mental disorders. The most common diagnoses are stress related states (e.g. burnout, depression, anxiety), and the prevalence as well as the increase are higher among women [4,5].

The modern concept of health is a complex interaction of environmental, organizational, and personal factors within the contexts and places that people live their lives. Thus broader environmental issues must be considered in matters of population health, something that has for example been reflected in studies of migrant populations where significant health effects by changing life environment have been demonstrated [6,7].

The salutogenic approach focuses on health factors (e.g. physical activity and healthy diet) as ways of maintaining good health [8]; access to nature is one such health factor that has received greater attention of late [9-11].

Some research on associations between nature and health has been based on evolutionary hypotheses, claiming that we have a genetic, inherent need for nature which by instinct makes us calm and less stressed in such settings [12-14].

Another hypothesis maintains that we are prone to mental fatigue, due to an overload of directed attention in our concentration demanding daily lives. In a natural setting, where spontaneous attention (fascination) is activated, this mental fatigue can be alleviated, allowing our brains to rest and recover [15,16].

Both these hypotheses link nature’s positive health outcome to reduced stress or mental fatigue, and there is good precedence for believing in the validity of these hypotheses. Since at least 50 years empirical studies have shown improved psychological wellbeing, reduced stress, and beneficial effects on pulse and blood pressure by contact with green spaces [17-20]. Data suggest that access and interaction with nature settings (e.g. parks, community gardens, urban greenways, forests, playing fields, and river corridors) has independent health effects and increase vitality and perceived general health [21,22]. Recently it was concluded that mental health is the realm of health that is probably most affected by green space in people’s neighbourhoods [23].

Apart from acting as a buffer between stressful life events and health, other mechanisms between nature and health have been considered. One such mechanism that has been suggested is physical activity. Access to nearby green areas is expected to increase physical activity and consequently also for example reduce levels of obesity [24-26].

Reduced socioeconomic health inequalities by access to green environments is another assumed mechanism for the link between nature and health [10], as well as increased social cohesion [27].

There is some consistency in the findings of access to nature as a positive factor for population health, but studies linking nature with health behaviour or health outcome has traditionally been mainly cross-sectional, and partly incoherent [28]. With few exceptions [29], there is also a lack of deeper exploration into what *kind* of nature is particularly beneficial to health. As an answer to this deficiency, attempts to assessing and categorizing certain nature characteristics have been considered. By storing such landscape assessments in Geographic Information Systems (GIS) a higher level of transparency is achieved.

GIS has emerged as an important computer-based tool in understanding spatial and temporal variation in human exposure to environmental and social determinants of health [30]. With GIS data specific qualities of nature can be mapped and integrated in epidemiological research in order to investigate the impact of *place* on health (hence referring to ‘place’ in the classic epidemiological triad of causation – person, place, time) [26,31,32].

In previous studies of connections between nature and health the effect of nature is often rather discrete. This is an expected phenomenon given the multifactorial background to many disorders. Hence spatial data may be considered as *complementary* in identifying health or risk factors. Complementary factors can be investigated by studying potential synergistic effects between theoretically plausible interactions, for example between “place” factors and “person” factors (referring to the triad of causation). Socioeconomic traits and physical activity are examples of person factors influencing health; in particular these factors are also suggested to correlate with the connections between green areas and population health [33-35].

Given that mental health may be affected by access to green spaces, and given that a few other person factors are suggested to interact and correlate with the connections between nature and health, a few questions arise. In this study the main research question is whether or not an inverse relationship exists between green qualities in the neighbourhood and development of mental disorder.

A secondary question is if there exist any synergistic mental health effects of nature (“place” factor) and a few life style related factors (“person” factors): financial stress, living conditions, and physical activity. Theoretically, those in financial stress or those living in apartments would benefit the most from nature, and the physically active population would have an even more beneficial effect on mental effect (than what would be expected as an outcome from the physical activity itself) if access to nature. We also aimed at scrutinizing any difference between each specific green quality, in terms of health effect and the synergistic effect.

## Methods

### Population

We analyzed a population from Southern Sweden aged 18–80 years in a follow-up study (1999/2000 to 2005). The Swedish registration system provides a personal identification number for every individual. This number can be used to link data from different registers, and can be used to follow each individual during the entire study period. In this study we also used register data for geo-coding of each individual. All other data were extracted from the surveys.

A health survey was distributed as a mailed questionnaire in 33 municipalities in the Scania region (a Southern Swedish province). The total sample comprised 24945 persons. Three mailed reminders and one reminder by telephone were used. In the baseline survey (1999/2000) answers were obtained from 13 604 (54.5%) respondents and 10 485 (77%) responded to the follow-up (2005). In this study we excluded the individuals living in larger city centres, due to lack of detailed landscape data in the evaluation of green qualities in these areas. The final cohort included 9230 persons.

The sampling was conducted with individuals, not households, as sampling units. We do not have data on the number of persons in the sample that belong to the same households, but it is assumedly a negligible number.

The initial public health survey was stratified to constitute a representation of the total population in Scania regarding gender, age, and education level [36]. At follow-up, women were slightly overrepresented (55.4% vs. 49.7% of non-responders) and fewer persons were born outside Sweden (9.2% vs. 14.8% among non-responders). There were also differences regarding unemployment (4.7% vs. 9.5% among non-responders), students (3.4% vs. 17.1% among non-responders), and low, middle, or high level non-manual workers (10.6%; 17.6% resp. 15.5% vs. 9.1%; 11.0% resp. 8.5% among non-responders). The responders were slightly more educated (38.1% had >13 years of education vs. 30.4% of non-responders). However, responders and non-responders had similar age (mean 49 years in both groups). Likewise, the level of physical activity was equivalent among responders and non-responders (low to moderate physical activity 78.8% resp. 77.8%).

There was a selective attrition based on mental health at baseline (21.7% of non-responders reported poor mental health at baseline compared to 17.6% of the responders).

Changes in residential addresses or in access to green qualities were not assessed for the non-responders.

In cases of extreme values (“outliers”) in data from 2005 those were controlled for and replaced with the values from the survey in 1999/2000. This was done for 60 cases concerning height, 10 cases concerning age (+ 5 years were added to 1999 value), and two cases concerning weight and ‘number of persons in the household’ respectively.

### Questionnaires

The survey and linking of register data were conducted in accordance with the Declaration of Helsinki and approved by the local committee of ethics (*Regionala Etikprövningsnämnden i Lund*, reference no. 2005–471).

The survey contained in total 106 questions on varied aspects of health. For the aim of this study we explored data on background variables – age, gender, economy, marital status, ethnicity, and education. Further on we extracted data on mental health (as measured by the General Health Questionnaire, GHQ-12) and data concerning habits of physical activity. Level of education was classified into four categories, close to the classification system of ISCED (International Standard Classification of Education) (UNESCO, 1997) [37]: 1) < 10 years at school, 2) 10–12 years at school, 3) vocational training, 4) university.

### Outcome variable, 2005

#### Mental health and general health questionnaire

There are several different versions of the self-administered General Health Questionnaire (GHQ), including GHQ-12 and GHQ-28. The GHQ-12 is a shortened 12-item version of the GHQ-28 [38], and is among the most widely used screening instruments for general mental health [39]. Prevalence of poor mental health is defined as reporting a problem in three or more of 12 questions in the GHQ-12 [40]. Each item (e.g. *“Have you, during the past few weeks, felt unhappy and depressed”*) is rated on a four-point Likert scale: 1) less than usual, 2) no more than usual, 3) rather more than usual, 4) much more than usual. Reporting a problem is defined as rating 3 or 4 on the item (scoring 0-0-1-1).

In general GHQ focuses on two main classes of phenomena: 1) inability to carry out one’s normal healthy functions; 2) emergence of new phenomena of distressing nature [41].

GHQ-12 has proven cross-cultural validity [42,43] and reliability with an internal consistency between 0.82 and 0.86 (Cronbach’s alpha) [44,45].

In this study GHQ-12 was used in Swedish and all items were applied. According to the validated syntax for GHQ12 a binary value was calculated for each individual 1999/2000 and 2005 – considered as having good mental health (interval 0–2) or not (interval 3–12).

### Exposure variables (place and person factors) and confounders, 1999

#### Green qualities (place factor)

Based on interview studies (focusing on how people perceive the landscape regarding preferences and habits), field studies, and inventories conducted in 1995–2005 in landscape architecture/environmental psychology, eight basic characteristics (or qualities) of the landscape were revealed (*Serene, Wild, Lush, Space, the Common, the Pleasure garden, Festive and Culture*) [46-50].

The green qualities have been suggested to be beneficial to health (hence they are sometimes denoted “recreational characters” or “recreational values”) and when used in previous epidemiological studies associations between access to these qualities and neighbourhood satisfaction as well as to physical activity have been demonstrated [26,51]. The green qualities have been used as a gold standard in a recently published epidemiological study [51], where area-aggregated assessments of the qualities demonstrated convergent as well as concurrent validity. However, though developed by experts in landscape planning, the qualities as such are not yet considered validated constructs.

To grasp features considered as healthy, resources for recreation have been classified and analyzed with GIS in former Swedish projects [52]. The National Land Survey of Sweden (Lantmäteriet) has within the European Union programme CORINE (Coordination of Information on the Environment) mapped the land and vegetation cover of Sweden into 58 classes, using 25 × 25 m grids [53]. With this data it was possible to establish objective definitions of the qualities that could be implemented using the GIS technique for five of the eight green qualities (Serene, Wild, Lush, Spacious, and Culture). These qualities were described and defined in GIS as below:

*Serene* – a place of peace, silence, and care. Sounds of wind, water, birds, and insects. No rubbish, no weeds, no disturbing people.GIS-criteria: broad-leaved forest, mixed forest, pastures, inland marshes, wet mires, other mires, water courses, lakes and ponds.*

*Wild* – a place of fascination with wild nature. Plants seem self-sown. Lichen and moss-grown rocks, old paths. GIS-criteria: Slopes more than 10°. Forest, thickets, bare rock, inland marshes, wet mires, other mires, water courses, lakes and ponds. Each >15 ha if >1 km from the city. **

*Lush* – a place rich in species. A room offering a variety of wild species and animals and plants. GIS-criteria: Mixed forest, marshes and mires, beaches, dunes, sand plains, bare rock. All registered “key biotopes”. Pasture land of regional interest. Biodiversity areas, bird biotopes. National parks

*Spacious* – a place offering a restful feeling of “entering another world”, a coherent whole, like a beech forest. GIS-criteria: Beaches, dunes, sand plains, bare rock, sparsely vegetated areas, burnt areas, natural grassland, moors and heath land, forest > 25 ha. Slopes > 10°. Farmland pointed out in a national plan. Coastal zone preservation. ***

*Culture* – the essence of human culture. A historical place offering fascination with the course of time. GIS-criteria: Non-urban parks. Farmland pointed out in a national plan. National interests of cultural preservation. Nature reservation areas. * Excluded areas: noise > 30 dB, artillery ranges. **Excluded areas: noise > 40 dB, <800 m to wind power aggregates. ***Excluded areas: noise >40 dB.

Only persons from rural or suburban areas, or smaller towns were included in this study (n = 9230), since the assessment of the green qualities could not be made objectively for inner city areas with available data. Hence individuals from the larger inner city areas (Malmö, Lund, Kristianstad, and Helsingborg) were excluded (n = 1245).

Residential geocodes were obtained for the participants. With the aid of those geocodes in combination with the GIS database the green qualities were included in our analysis. We assessed for each respondent the *presence/absence* (regardless of amount/area) of each of the five qualities within 300 m from the centre of the property at the geocoded residential address. We assessed either *amount* of green qualities (zero to five), or access or not to each single quality respectively (i.e. *access to serene or not, access to wild or not, etc.*)

Concerning the chosen distance of 300 m it can be commented that in Scandinavia a common average distance to urban green areas is 300 m [54]. In addition 300 m has previously been estimated as a crucial limit for people to exploit green spaces for recreational purposes and it is believed to represent rather well a person’s recreation area in his/her neighbourhood [17,26]. A distance of 300–400 m is often reported as the threshold after which use starts to decline rapidly [17,20,55].

#### Physical activity (person factor)

There are varied approaches to measuring physical activity [56]. In this study we dichotomized the population according to a single question concerned with leisure-time physical activity – *“How often are you physically active of perform exercise during you leisure time? Excluding domestic work” (Response alternatives: 1) Sedentary 2) Moderate physical activity 3)Regular exercise 4) Regular advanced exercise)*. Low to moderate leisure-time physical activity was defined in this study as responding 1 or 2 (n = 6811; 78.8%), and regular leisure-time physical activity as responding 3 or 4 (n = 1838; 21.1%).

To study the potentially increased mental health effect of physical activity and access to nature, or any particular kind of nature, interaction-variables were created between low or regular physical activity respectively and either access to the green qualities or not, or access to each single quality (Wild, Lush, Serene, Culture, or Space) or not.

#### Financial stress and living conditions (person factors)

Classification in three groups of *financial stress* was based on data about having troubles paying bills (*1)every month 2)every second month 3)it occurs rarely 4)never*). Persons reporting troubles often (i.e. responding 1 or 2) were classified as financially stressed, reporting problems rarely (i.e. 3) as slightly financially stressed, and never troubled (4) as not financially stressed. These groups were used to study potential interaction effects between financial stress and access to the green qualities (in aspects of amount or particular quality).

Concerning living arrangements and form of housing the classification was constructed in accordance with living in detached houses or terrace-houses (group 1), living in a block of flats (group 2), or other living forms (group 3). Again those groups were used to study any interaction effect with the green qualities; those living in a block of flats were assumed to benefit the most of access to nature.

#### Potential confounders

All persons born in a country other than Sweden were merged into one single category. Hence the categories of *country of origin* are “Sweden” or “other” (n = 810; 9.1%).

*Marital status* was classified into two groups (cohabiting or not) according to four response alternatives – 1)*married or cohabitant* 2)*unmarried* 3)*divorced* 4)*widow/er*. Hence any other response than *1* was merged into one group, considered as living alone (n = 2237; 25.3%).

### Statistical analysis

Adjusted for baseline mental health, crude odds ratios (OR) and 95% confidence intervals (CI) were calculated in order to analyze associations between different exposure variables in 1999, and the outcome, mental health in 2005. Thereafter multivariate logistic analyses were performed. Apart from the exposure variables mentioned in the previous section, *mental health 1999* and *age* were included as confounders.

In a similar survey conducted in the same population of Southern Sweden, multilevel analyses of green qualities aggregated to 1000 square metres did not change results compared to single level analyses, suggesting negligible clustering-effect even for much smaller areas than municipalities [51]. The same negligible cluster-effect has also been found in other studies of relationship between behaviour, health outcome and green space [57]. For the data from 1999/2000 the non-clustering-effect was also tested empirically. Thus in this study we fitted single level regression models to the data.

In the preliminary analyses we found no support for any interaction effects between financial stress and green qualities, nor for living arrangements and green qualities. Since a pattern appeared for physical activity and the green qualities we decided to focus on that in the following analyses. The effect of the interaction variable, constructed from physical activity and access to green qualities (both quantitatively and qualitatively), was explored by logistic regression analysis concerning the association and OR for mental health outcome. Any significance of positive departure from additivity of effects by the interaction variable was calculated by relative excess risk due to interaction (RERI) [58,59].

Given the central focus on setting and living environment the analyses were restricted to those who did not experience any change in environment between base-line and follow up (n = 7549). This was to keep the access to green qualities constant between the occasions and hence reduce the potential effect on mental health that may be expected from a move, and that is not attributable to the environment itself.

All analyses were conducted using SPSS 18.0 for Windows (SPSS Inc, Chicago, Illinois, USA). The statistical significance level was set to p-value < 0.05 and 95% CI for mean differences and OR.

## Results

### Prevalences

Table 1 demonstrates that the green qualities were rather equally distributed between the genders. The proportion of affected mental health status, according to GHQ 12, was larger among women, and we decided to run the analyses separately by gender. The proportion of persons born outside Sweden was almost the same for men and women. In general men were more often cohabiting and experienced slightly less financial stress.

**Table 1:** Prevalence of demographic, recreational environment, physical activity, and mental health variables

	**Men**		**Women**		**Total**	
	**N**	**%**	**N**	**%**	**N**	**%**
**Recreational characters 1999:**						
**Wild**	145	3.6	176	3.5	320	3.6
**Space**	478	12.0	523	10.5	1001	11.2
**Serene**	244	6.1	313	6.3	558	6.2
**Culture**	981	24.5	1171	23.6	2154	24.0
**Lush**	1116	27.9	1291	26.0	2408	26.9
**Number of recreational characters 1999:**						
**0**	2220	55.5	2876	57.9	5098	56.8
**1**	1073	26.8	1284	25.9	2359	26.3
**2**	358	9.0	415	8.4	774	8.6
**3**	223	5.6	221	4.5	444	5.0
**4**	109	2.7	153	3.1	262	2.9
**5**	14	0.4	17	0.3	31	0.3
**Poor mental health 1999 (GHQ12)**	498	13.1	883	19.0	1424	16.4
**Physical activity 1999:**						
**Sedentary**	525	13.6	673	14.1	1198	13.9
**Moderate activity**	2477	64.1	3133	65.5	5613	64.9
**Regular exercise**	732	18.9	922	19.3	1654	19.1
**Regular advanced exercise**	129	3.3	55	1.1	184	2.1
**Country of origin (1999):**						
** - other than Sweden**	366	9.2	444	9.0	810	9.1
**Cohabiting :**						
** - No**	938	23.8	1299	26.5	2237	25.3
**Financial stress :**						
** -Stressed**	287	7.3	459	9.5	746	8.5
** -Slightly stressed**	669	16.7	895	18.5	1564	17.9
** -Not stressed**	2957	75.6	3485	72.0	6445	73.6
**Mean age (years)**	50.8		50.0		50.1	

N = 7549 (persons who had moved between the occasions excluded).

### Crude odds ratio, adjusted for mental health 1999/2000

Table 2 illustrates that for both genders a poor mental health status at baseline is a clear significant risk factor for poor mental health at follow-up. For men there was a tendency, though not significant, of decreased risk for poor mental health at follow-up with increasing numbers of green qualities. Moderate or even more regular physical activity significantly decreased the risk for poor mental health at follow-up. Moderate to severe financial stress had significant impact on the risk for poor mental health for both genders. Not cohabiting imposed a significantly increased risk for poor mental health for both men and women. Increasing age seemed to decrease the risk for mental health problems. Among persons born outside Sweden the risk was higher for affected mental health status, with a stronger effect among men. Regarding the interaction variables (constructed from access to each green quality respectively and physical activity, or amount of green qualities and physical activity) there was an effect from physical activity and Serene and/or physical activity and Space. There was no certain effect to be seen for amount of green qualities and physical activity; however this variable was contained in the multivariate analysis because of its relevance for exploring the amount-effect of access to green qualities.

**Table 2:** Crude odds ratios (OR) and confidence intervals (CI), adjusted for mental health 1999, of risk for poor mental health in 2005 in relation to

	**Men**		**Women**	
	N	Crude OR, (95% CI)	N	Crude OR, (95% CI)
**Poor mental health 1999**	364	5.3(4.1–6.8)	633	3.7 (3.0–4.5)
**Access to Serene 1999**	384	0.9 (0.6–1.5)	670	0.8 (0.6–1.2)
**Access to Wild 1999**	384	0.6 (0.3–1.2)	670	1.4 (0.9–2.2)
**Access to space 1999**	384	0.8 (0.6–1.2)	670	0.9 (0.6–1.2)
**Access to culture 1999**	384	0.8 (0.6–1.1)	670	1.0 (0.8–1.3)
**Access to lush 1999**	384	0.9 (0.7–1.1)	670	0.9 (0.7–1.0)
**Access to recreational values 1999**				
**1 vs zero**	1030	0.9 (0.7–1.1)	1198	1.0 (0.8–1.2)
**2 vs zero**	339	0.8 (0.5–1.2)	390	0.9 (0.6–1.3)
**3 vs zero**	213	1.0 (0.6–1.6)	207	1.0 (0.6–1.5)
**4 vs zero**	98	0.7 (0.3–1.6)	141	0.8 (0.4–1.5)
**5 vs zero**	13	0.3 (0.04–2.8)	14	1.5 (0.4–5.6)
**Physical activity 1999**				
**Little vs sedentary**	2353	0.7 (0.5–1.0)	2936	1.0 (0.8–1.3)
**Regular exercise vs sed**	694	0.7 (0.5–1.1)	869	0.9 (0.6–1.2)
**Advanced exer vs sed**	124	0.9 (0.4–1.9)	52	0.2 (0.05–0.9)
**Financial stress 1999**				
**Severe vs none**	274	2.9 (2.1–4.2)	436	2.2 (1.7–2.9)
**Moderate vs none**	629	1.5 (1.2–2.1)	842	1.5 (1.2–1.9)
**Not cohabiting vs cohabiting (1999)**	469	1.4 (1.1–1.8)	844	1.1 (0.9–1.4)
**Age**				
**39–51 vs younger**	921	0.8 (0.6–1.0)	1213	0.8 (0.7–1.0)
**52–62 vs 18–38**	967	0.5(0.4–0.7)	1089	0.7(0.5–0.8)
**63–81 vs 18–38**	908	0.6(0.4–0.9)	1017	0.6(0.5–0.8)
**Country of origin**				
**–Other than Sweden**	475	2.5 (1.8–3.4)	848	1.2 (0.9–1.6)
**Interaction variables, 1999:**				
Access to ser and active*		0.3(0.04–2.5)		0.2(0.06–0.9)
Access to ser and passive*		1.1 (0.7–1.8)		1.0 (0.6–1.5)
Not access to ser and active*		1.1 (0.8–1.4)		0.9 (0.7–1.2)
Access to wild and active*		1.4(0.4–5.3)		1.1 (0.4–3.3)
Access to wild and passive*		1.0 (0.8–1.3)		0.9 (0.7–1.1)
Not access to wild and active*		0.4 (0.2–1.1)		1.4 (0.8–2.4)
Access to space and active*		0.9(0.4–2.3)		0.3(0.1–0.9)
Access to space and passive*		0.8 (0.5–1.3)		1.0 (0.7–1.4)
Not access to space and active*		1.0 (0.8–1.4)		1.0 (0.8–1.2)
Access to culture and active*		0.8(0.5–1.5)		0.8(0.5–1.2)
Access to culture and passive*		0.8 (0.6–1.2)		1.1 (0.9–1.4)
Not access to culture and active*		1.1 (0.8–1.4)		0.9 (0.7–1.2)
Access to lush and active*		0.8(0.5–1.4)		0.8(0.5–1.3)
Access to lush and passive*		0.9 (0.7–1.3)		0.8 (0.6–1.0)
Not access to lush and active*		1.1 (0.8–1.5)		0.9 (0.7–1.1)
Access to 1 or more recr values and active**		0.8(0.5–1.3)		0.8(0.6–1.1)
Access to 1 or more recr values and passive**		0.9 (0.7–1.1)		1.0 (0.8–1.2)
No access to recr values and active**		1.1 (0.8–1.5)		0.9 (0.7–1.2)

N = 7549 (persons who had moved between the occasions excluded).

### Adjusted odds ratios

**Figure 1:**
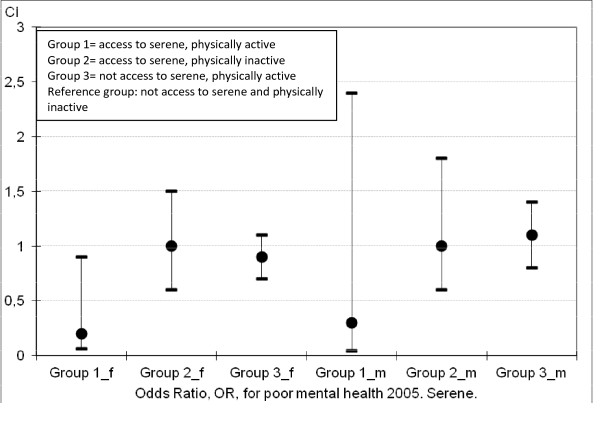
**Risk for poor mental health 2005, female (f) and male (m) participants.** OR and CI for interaction-effect between physical activity and the green quality serene.

**Figure 2:**
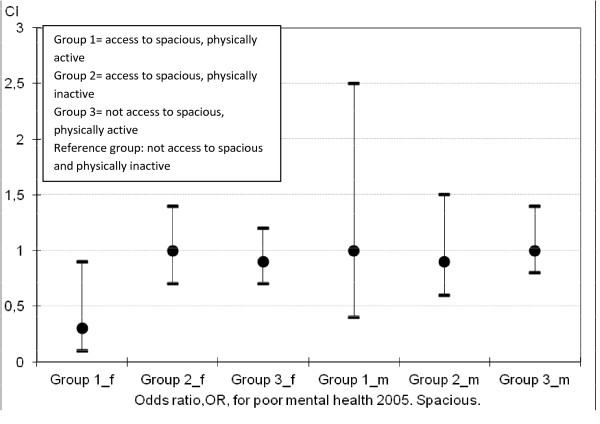
**Risk for poor mental health 2005, female (f) and male (m) participants.** OR and CI for interaction-effect between physical activity and the green quality spacious.

Table 3 demonstrates that, when adjusted for financial stress, cohabitation status, country of origin, age, and mental health state at baseline, the green qualities without interaction have no significant impact on mental health at follow-up. However, as is shown in Figures 1 and 2, the interactive effect of physical activity and access to the green qualities Serene or Space significantly reduces the risk for poor mental health at follow-up (OR = 0.2 and 0.3 respectively) among women. The positive departure from additivity effect was significant for Serene (*p* = 0.04, RERI = −0.62, 95% *CI* = −1.21 to −0.03). For men OR was 0.3 for the interaction variable containing Serene, though the positive departure from additivity was not significant (*p* = 0.09, RERI = −0.79, 95% *CI* = −0.79 to 0.12). Regarding the interaction variable including Space no significant effect was seen for men, and the positive departure from additivity effect was borderline significant for women (*p* = 0.05, RERI = −0.57, 95% *CI* = −1.13 to −0.01). The ORs for the remaining interaction variables, including Wild, Culture, and Lush, were all non-significant.

**Table 3:** Multiple logistic regression. Adjusted multivariate odds ratios (OR), 95% confidence intervals (CI), and p-tests for risk of poor mental health in 2005

	**Men**			**Women**		
	OR	CI	p	OR	CI	P
**Slight financial stress**^**a**^	1.4	1.0–1.9	0.039	1.3	1.1–1.7	0.015
**Severe financial stress**^**a**^	2.3	1.6–3.4	<0.001	2.0	1.5–2.7	<0.001
**Not cohabiting**^**b**^	1.3	1.0–1.7	0.089	1.0	0.8–1.3	0.67
**Born outside Sweden**^**c**^	2.1	1.5–2.9	<0.001	1.1	0.8–1.5	0.63
**Age**	1.0	0.98–1.0	0.032	1.0	0.98–0.99	<0.001
**Poor mental health 1999**^**d**^	4.2	3.2–5.5	<0.0001	3.2	2.6–3.9	<0.001
**Access to serene**	0.9	0.5–1.6	0.77	0.8	0.5–1.2	0.29
**Access to space**	1.1	0.7–1.6	0.74	1.1	0.8–1.6	0.54
**Access to 1 or more recr values**	0.9	0.6–1.2	0.49	0.9	0.7–1.2	0.54
**Physical activity**						
**Little vs sedentary**	0.9	0.7–1.3	0.65	1.1	0.8–1.4	0.47
**Regular vs sedentary**	0.9	0.6–1.4	0.79	0.9	0.6–1.3	0.54
**Advanced vs sedentary**	0.9	0.5–1.6	0.77	0.2	0.04–0.83	0.027
**Interaction variables:**						
**Access to serene and physically active**^**e**^	0.3	0.04–2.4	0.25	0.2	0.06–0.9	0.05
**Access to space and physically active**^**e**^	1.0	0.4–2.5	0.96	0.3	0.1–0.9	0.045
**Access to 1 or more recr values & physically active**^**e**^	0.9	0.6–1.4	0.66	0.8	0.6–1.1	0.21

N = 7549 (persons who had moved between the occasions excluded).

^a^ vs no financial stress.

^b^vs cohabiting.

^c^vs born in Sweden.

^d^vs good mental health 1999.

^e^interaction variable. Reference category: no access to the recreational character and physically inactive.

In the adjusted model the effect of financial stress was decreased, especially for men with poor economy (OR = 2.3, compared to crude OR = 4.6). The effect of cohabiting on mental health outcome was also decreased to no effect among women, and a non significant effect for men (OR = 1.3). The adjusted model decreased the impact of country of origin as well, OR = 2.1 for men, and no association for women. Concerning physical activity, the pattern of only advanced exercise for women as a significant risk reducer remained in the adjusted model. The *amount* of green qualities was still without significant effect, also when included in the interaction variable.

## Discussion

In this cohort study we did not find a simple inverse relationship between green qualities in the neighbourhood and development of mental disorder.

However, we found a synergistic mental health effect through interaction between certain green qualities and physical activity. The risk of having poor mental health at follow-up decreased 80% if having access to Serene and being physically active and 70% if access to Space and physically active, compared to not having access to either of these qualities and being physically inactive. These effects were statistically significant for women, but not for men. However, the tendencies were the same for men.

Regarding the other three green qualities as well as amount of qualities no statistically certain synergistic effects were found.

The strength of this study is the longitudinal perspective in a relatively large cohort. Data were achieved from a health survey that was broad and contained a validated instrument (GHQ12) for measuring mental health. The extensiveness of the questionnaire made detailed confounding control possible.

Another advantage was the objective measures of nature by the storage of predefined green qualities in GIS. This enabled transparent environmental neighbourhood assessments that could be correlated to estimates of the participants’ mental health. Due to technical restrictions we were only able to assess five of the original eight qualities, but among these five, the three qualities that have been considered the most important in aspects of stress relief (Wild, Serene, and Space) were included [17,46].

Our study is geographically restricted to Southern Sweden. The particular area studied is in general quite limited in aspects of natural resources and green qualities; the average amount of green qualities in the population was small (μ = 0.72 at baseline and 0.71 at follow-up). As in any epidemiological study we must also be aware of the risk for neglected confounders. Other limitations include that the exposure variable financial stress was constructed from one single question about problems with paying bills. However, this construct has been used in former studies, and has also been found to correlate with poor self-rated health [26,60]. We were also dependent on existing data from the survey on physical activity, and the measure is not validated.

The selective attrition on the basis of mental health at baseline poses a risk for selection bias. However, this should not affect the relative risk estimate, since we assume that loss to follow-up applied equally to the exposed (to green qualities and physical activity) and non-exposed group [61]. There seemed to be no selection bias concerning level of physical activity.

The statistical significance levels found for the additive effect of Serene and Spacious nature to physical activity among women, were 0.050 and 0.045 respectively, thus the risk for false positive results must be considered. However, the *tendency* of a measurable effect for certain qualities seemed to be consistent.

Regarding the indicated gender difference concerning the benefits from surrounding nature the mechanism is obscure. It must be acknowledged that the effect trends were the same for women and men; only the significance was weaker for men. Theoretically possible explanations for plausible gender discrepancies might be varied use of nature between the sexes, or variance in response to stress, mental issues, and necessary restorative experiences. Gender differences in perceiving and experiencing natural landscapes may also exist. From brain-imaging studies gender-related differences in the neural correlates of aesthetic preference have been found [62].

A common assumption in research on nature and health has been “the more the merrier”, in the sense that you would presume more nature to result in an increased health outcome. We did not find any support for this assumption in our study, but it suggested that the specific *quality* of greenery might be more important than quantity, and that this quality can actually be specified. This is in line with former studies, which have shown that the quantity as well as the quality of neighbourhood greenspace seem relevant with regard to health [29].

Given the multifactorial background to mental disorders and other non-communicable diseases the concept of synergies and interaction effects is interesting. This potentially also allows for detection of influential factors with otherwise too small effect sizes. Physical activity is increasingly being recognized as not only beneficial to physical, but also to mental health [63-65]. It is noteworthy that *moderator variables* have previously been demonstrated to influence the nature and magnitude of the relationship between exercise and different mental health outcomes [66]. In animal studies so called “enriched environments”, where exercise is regarded as one of the components, have been found to promote neurogenesis and enhance memory functions [67].

## Conclusions

We did not find a direct connection between green qualities and mental health in this study, but it does not necessarily mean that this connection does not exist. As previously mentioned the studied area is rather low in landscape diversity, hence the studied differences in access become rather subtle, something that might require larger power of the study in order to detect significant effects. There is ongoing research on the qualities to strengthen their validity and consequently applicability. More stringent precision and validations of the methods would hypothetically enable the revealing of significant connections between certain green qualities and certain health outcome. In this perspective it is interesting that we *did* find tendencies of health effects from green qualities, given the non-validated landscape data and the sub-optimal geographic area in this study. This stresses the relevance of further studies since a validated landscape assessment tool would be an utterly important method for landscape planners and population health workers.

A considerable body of research has shown links between health and nature, but until date the implications for policy and decision making, whether it concerns population health issues or landscape planning, have been scarce. In this study we have found that in interaction with physical activity the qualities Serene and Space have some risk-reducing effect on mental health disorders for women, an impact that seems to overshadow the mere amount of nature. This in turn might be considered in the practical design and management of everyday environments. Notwithstanding some limitations and restrictions we believe that this study may bring us closer to positive and efficient implications and use of green spaces in relation to population health.

## Competing interests

The authors declare that they have no competing interest.

## Authors’ contributions

All authors participated in the design of the study for this report. MA analyzed most of the data and wrote the manuscript. POÖ was principal investigator and revised the manuscript. JB was main supporter in statistical analyses and made the RERI analyses, and revised the manuscript. PG was responsible of the hypothesis making and was involved in revising the manuscript. EÄ was responsible of the landscape assessment analyses and was involved in revising the manuscript. PW was responsible of the general study agenda and was involved in revising the manuscript. All authors read and approved the final manuscript.

## Pre-publication history

The pre-publication history for this paper can be accessed here:

http://www.biomedcentral.com/1471-2458/12/337/prepub
